# Paramedic. *noun.*

**DOI:** 10.29045/14784726.2019.09.4.2.1

**Published:** 2019-09-01

**Authors:** Georgette Eaton

**Affiliations:** On behalf of the Board of the College of Paramedics

*‘A paramedic works autonomously as a generalist clinician across a range of healthcare settings, usually in emergency, primary or urgent care. They may also specialise in clinical practice, education, leadership or research.’*

*—College of Paramedics (2019)*

To patients and members of the public, our profession is often synonymous with ambulance services, blue lights and sirens; delivering emergency care in green uniforms. Dictionary definitions (Table 1) only confirm this supposition, placing the paramedic at the centre of the delivery of emergency care at the scene of an accident or emergency.

**Table 1.** Dictionary definitions of a paramedic.

Dictionary	Definition
Cambridge Dictionary (2019)	A person who is trained to do medical work, especially in an emergency, but who is not a doctor or nurse.
College of Paramedics (2018)	A paramedic works autonomously as a generalist clinician across a range of healthcare settings, usually in emergency, primary or urgent care. They may also specialise in clinical practice, education, leadership or research.
Collins Dictionary (2019)	A paramedic is: 1: a person whose training is similar to that of a nurse and who helps to do medical work; 2: a member of an ambulance crew trained in a number of life-saving skills, including infusion and cardiac care.
Dictionary.com (2019)	A person who is trained to assist a physician or to give first aid or other healthcare in the absence of a physician, often as part of a police, rescue or firefighting squad.
Lexico (2019)	A person trained to give emergency medical care to people who are seriously ill with the aim of stabilising them before they are taken to hospital.
Macmillan Dictionary (2019)	Someone who is trained to give medical treatment to people at the place where an accident has happened.
Merriam Webster (2019)	1: A person who works in a health field in an auxiliary capacity to a physician (as by giving injections and taking X-rays). 2: A specially trained medical technician licensed to provide a wide range of emergency services (such as defibrillation and the intravenous administration of drugs) before or during transportation to a hospital – compare EMT.
Oxford English Dictionary (1966)	One trained to provide specialised emergency medical care.
Urban Dictionary (2004)	An absolute hero. Largely unappreciated by the general population until they are gasping for their last breath after 25 pints of Stella and a kicking par excellance [sic] on a Friday night.

This is not wrong. A large percentage of paramedics do work in ambulance services. We do drive under emergency conditions, we do deliver emergency care and we do wear (varying shades of) green uniform. However, times are changing and ‘*not all paramedics wear green*’.

In 2019, these definitions do not represent the *modern paramedic*. They give little to no insight into the range of work that paramedics can, and do, undertake. In a little over 20 years, our profession has become professionally registered, has crossed the threshold into the academy and is working in healthcare environments previously exclusive to medicine. Indeed, not all paramedics are clinical, with a growing number working in research, education and senior leadership roles. The Board of Trustees at the College of Paramedics recognised that these ‘long-standing’ definitions were no longer fit for purpose – that they no longer defined the paramedic within the UK. We were therefore tasked with modernising the definition, ensuring it represented all paramedics, in all settings.

However, we cannot escape the noun under which we are titled. Aronson (2017) has previously outlined what is meant by our title: It is the Greek word πᾰρά which gives the English prefix of ‘para’, meaning alongside, beside, by or near. Inherent in our title, is that our work will always be alongside medicine. The crucial element here is *alongside*. Not as an auxiliary, assistant or replacement of – but alongside. *Together.*

Then what is our unique selling point? Nurses, physiotherapists, radiographers, speech and language therapists (SALTs) and physician associates (PAs) (to name a few) all work alongside medicine. All are professionally registered (with PA regulation on the horizon), and physiotherapists, SALTs and radiographers find themselves under the ‘Allied Health Professional’ umbrella, together with paramedics. Our difference comes with our history. For our profession, working *alongside* medicine has meant that we have always needed to be generalist in our clinical practice. Even the 1980s ‘paramedic’ was required to work independently from hospital-based doctors, working within a retrieval-based ambulance service. This is a key point in differentiation – the core role of the paramedic still requires that we are capable of operating as a generalist, in the sense that we deliver care to patients of all ages, who may present with a broad range of conditions, with varying complexity and within a wide range of environments. All this, autonomously, from the point of registration.

Yet the current, popularised definitions of a ‘paramedic’ do not capture this. We continue to be defined by the historical settings of our work, and often suffer a loss of professional title to become ‘ambulance drivers’. In some definitions, we are also defined by what we are not, which is even less informative. It is difficult to hinge a definition on one setting in which paramedics work clinically, given our ability to work in many clinical settings, growing within different specialities. The paramedic definition should not be limited to one clinical setting. Equally, listing the current clinical environments may only seek to act as a restriction. The definition must be contemporaneous, and also future proof. It also needs to have clarity regarding the type of clinical work we do undertake. Balancing being too broad, or too restrictive, the definition needs to outline the variety of clinical settings we work within. This is not only for us and other healthcare professionals; how can future generations of potential paramedics be inspired if the dictionary definition of our role does not actually inform as to what we do?

The current dictionary definitions also conflate our professional title across different countries. The word ‘paramedic’ is a protected title and it is registered within the United Kingdom, Australia, Canada and certain states within the United States. However, it is apparent that one definition does not fit all registered paramedics, and the scope of practice, level of autonomy and settings in which they work varies across these countries. Such conflation only serves to confuse members of the public and does little to explain the meaning of the term.

In addition, each of the definitions focus on the clinical capabilities of the paramedic. There are four pillars within healthcare – clinical practice, education, leadership and research – and none of these definitions observe these aspects of our work. Indeed, neither do the definitions of other healthcare professionals. It was important to the Board that this definition represented all paramedics, in all settings. It was also important to shift the synonymous nature for patients and the public reading this definition, outlining that paramedics do not only treat patients, but also teach those joining the profession and continue to develop the role through research and leadership. Whatever we are doing now, we know that paramedics will continue to work in these settings in 5, 10 and 20 years’ time. The work we undertake will (hopefully) continue to evolve and develop, but our presence within each pillar will always remain.

We continue to move further away from the historical definitions that focused on where we practise, but perhaps not how we practise. It is time that our profession was defined by those who practise it, and that patients, members of the public and other healthcare professionals can both understand and appreciate that our profession is defined by more than the green uniform, an ambulance and blue lights.
